# Analyzing drivers’ perceived service quality of variable message signs (VMS)

**DOI:** 10.1371/journal.pone.0239394

**Published:** 2020-10-21

**Authors:** Zhuanglin Ma, Mingjie Luo, Steven I-Jy Chien, Dawei Hu, Xue Zhao

**Affiliations:** 1 College of Transportation Engineering, Chang’an University, Xi’an, China; 2 John A. Reif, Jr. Department of Civil and Environmental Engineering, New Jersey Institute of Technology, Newark, NJ, United States of America; 3 School of Science, Xi’an Shiyou University, Xi’an, China; South China University of Technology, CHINA

## Abstract

Recent advance in VMS technology has made it viable to ease traffic congestion and improve road traffic efficiency. However, the drivers’ low compliance with the posted information may limit its performance to ease traffic congestion and improve traffic safety. This paper explores drivers’ attitude to the service quality of VMS system resulted from the identified predominant influencing factors. A questionnaire is developed and used for surveying 9,600 drivers in Beijing, China. The collected data are analyzed with a multiple indicators and multiple causes (MIMIC) model considering different driver categories (e.g., private car driver, office car driver, taxi driver). The results show that the causal relationships between latent variables and socio-demographic characteristic is significant. Driving frequency, attitude towards contents of VMS, drivers’ decision-making and the effectiveness of VMS message can directly and indirectly affect driver’s perceived quality of service. The attitude towards formats of VMS indirectly affect their QoS resulting from the effectiveness of VMS message, while there is no indirect impact for taxi drivers. Besides, the drivers’ decision-making directly affects the perceived quality of service for private car drivers and office car drivers, but there is no impact for taxi drivers. The findings of this study can provide guidance and reference for urban authorities to perform the relevant actions required to meet user expectations.

## Introduction

Variable message sign (VMS), also known as changeable message sign (CMS) or dynamic message sign (DMS), has been applied to disseminate guidance information (e.g., traffic information, road condition, parking and public transport information, etc.) to road users. The VMS information plays a significant role to guide vehicles away from a congested route caused by recurring and non-recurring events. Unlike highway advisory radios (HAR) and smartphones, VMS can be used without any equipment on the driver’s side, hence it is an effective traffic management measure for alleviating traffic congestion caused by roadworks, special events, incidents, effectively distributing traffic flow and reducing traffic safety risks [1–4].

While VMS have been widely used for disseminating road information, the problems of instantaneous information, unreasonable layout and drivers’ poor compliance to the VMS information still exist, which leads to a decrease in traffic induction efficiency and the extent of their capabilities for improving network performance under incident/congested situations has not been exploited sufficiently [5]. Therefore, this paper aims to study how to improve the driver’s obedience to the VMS information.

Because VMS panels are often costly to design, install and maintain, drivers’ perception becomes an important indicator of potential demand and is often cited by agencies as a means to justify the implementation of a new system or improve existing ones [6]. Whether drivers can comply with the VMS information, and whether the VMS effectively distributing traffic flow depend largely on the drivers’ perception and attitude of the VMS information. Therefore, it is essential to understand drivers’ perception of VMS system and identify the predominant factors influencing drivers’ perceived quality of service.

Most previous studies were focusing on the layout and setting of VMS, while there was a dearth of research on evaluating the service quality of VMS system from the perspective of driver perception. The purpose of this study is to explore drivers’ attitude to service quality of VMS system and identify the predominant influencing factors, thereby allowing agencies to undertake appropriate measures to further improve drivers’ compliance with information provided by VMS.

One contribution is that this paper is the first study to develop a holistic model which can be applicable to three types of drivers, and use a multiple indicators and multiple causes (MIMIC) model to investigate the factors influencing drivers’ attitude to service quality of VMS system according to different driver categories in order to explore the differences between different drivers. Additionally, from a practical point of view, this study will provide guidance and reference for other urban authorities to perform the relevant actions required to meet user expectations.

## Literature review

### Development status of VMS

At present, with the rapid development of modern communication technology and Internet technology, VMS has been widely used in China. VMS is one of the main means to ease traffic congestion, ensure vehicle safety and improve road traffic efficiency. Its main function is to display various traffic-related guidance information in time according to the collected real-time traffic information and the command and dispatch instructions of traffic management departments, which induces drivers to make correct route decisions and makes the traffic network evenly distributed, thus improving road traffic efficiency and reducing the travel time of travelers [7].

However, there are still some gaps in layout, operation and management compared with developed countries. Problems such as unreasonable layout of VMS, poor accuracy of VMS information, inconsistent display of information and low driver compliance still exist [8]. In addition, with the installation of VMS in more cities, the research on VMS has gradually become a craze. The research and application of VMS mainly focus on route decision, information display and layout setting. However, it is rare to study the application and optimization of VMS system and the service effect of VMS. Therefore, the research and construction of VMS still have huge development potential and wide space.

### Factors affecting driver’s perceived quality of service

A high-quality traffic information system, from the road users’ perspective, should provide accurate, reliable and easy-to-understand traffic information, which enables drivers to make correct decisions on departure time as well as route choice [6]. In addition, the displayed contents and formats must meet the driver’s expectations. One might therefore refer to the constructs “attitude towards formats of VMS”, “attitude towards contents of VMS”, “driver decision-making” and “effectiveness of VMS message”, as determinants as they directly or indirectly explain driver’s perceived quality of service [6, 9, 10].

Some studies have used the drivers’ decision to change route upon receiving a set of traffic information provided by the VMS as the sole surrogate for perceived system quality to evaluate the effect of the VMS [3, 11, 12]. However, this measure does not consider the many externalities that can also affect driver’s perceived quality of service, including the perceived accuracy and reliability of traffic information, and drivers’ attitudes towards the attributes of VMS. By studying the drivers’ attitudes towards the attributes of VMS, the perceived quality of service system can then be estimated. Accordingly, Zhang and Li (2018) in the aspect of public transport released by VMS are the main factors affecting the driver’s route decision [13]. Li (2010) found that the driver’s route decision would be significantly affected by the contents and formats of VMS [10]. In addition, some studies have provided evidence that the effectiveness of VMS message can significantly affects driver’s perceived quality of service. For example, Xu et al. (2010) suggested that effectiveness of VMS message could significantly affect drivers’ acceptance of the traffic information [14]. Khoo and Ong (2013) also concluded that a better perception of ATIS system effectiveness, accuracy and reliability can well indicate a high perceived quality of service [6].

However, some studies indicate that the socio-economic attributes and travel characteristics are also considered as important factors for evaluating service quality of VMS. Peeta and Ramos (2006) found that the driver’s reaction and behavior under the traffic information provided by VMS were influenced by the driver’s socio-economic attributes and travel characteristics [5]. Zhong et al (2012) suggested that the driver’s route choice behavior is related to age, driving age, income and road network familiarity [15]. Cao et al (2014) indicated that driver’s route change frequency was affected by gender, educational background, vehicle type and travel activity [16].

### Analysis methods for measuring drivers’ perceived quality of devices

Previous scholars mainly used questionnaire survey to evaluate the service quality of VMS. One is to investigate the traffic operation effect, for example, how installation of VMS along the road affects the average driving speed or average delay? The evaluation does not consider the transportation users, such as how users perceive the service quality of these devices or the satisfaction of users with the services they receive [2, 17]. The second is a sample survey of drivers, which investigates users’ satisfaction with the traffic services provided by VMS based on a sample survey of drivers by asking users’ opinions on the traffic service information they use [18, 19]. However, Lee et al (2004) pointed out that it is difficult to describe the survey results quantitatively and objectively and to analyze the key factors affecting users’ perception of ATIS service quality only by using surveys. Accordingly, Lee et al. (2005) proposed a method based on fuzzy aggregation to evaluate VMS service quality [20]; Shao et al (2010) evaluates VMS information service based on the multinomial Logit model. However, little of these methods have been used to develop a structure model to exploring the latent relationship between variables [21].

To make up for this deficiency, the structural equation modelling (SEM) has been used to evaluate perceived service quality in the aspect of public transport [22, 23], rail transit [24], water transit [25], as well as traffic information system [26]. However, SEM is difficult to deal with individual characteristics because they affect perceived quality as casual variables rather than indicator variables. A multiple indicators and multiple causes (MIMIC) model can cope with casual variables (i.e. gender, age, et al.) and indicator variables (i.e. service quality) concurrently and capture the heterogeneous individual characteristics in different groups of individual characteristics [27]. Hence, MIMIC has been widely utilized to measure service quality and satisfaction [28]. To explore the relationship between variables, and the relationship between socio-demographic variables and potential variables, a MIMIC is therefore developed in this paper to study the factors affecting driver’s perceived quality.

## Methodology

This section first describes the stated preference (SP) questionnaire in detail, and introduces the specific process of sample investigation, then sets out the basic principle of MIMIC model, and finally puts forward the corresponding research hypothesis according to the research content.

### Ethics statement

Ethics statement was approved by the Beijing Traffic Management Bureau, Beijing, China. The study was also approved by the College of Transportation Engineering of Chang’an Univeristy. All respondents provided written informed consent.

### Questionnaire describing

A stated preference (SP) questionnaire survey is designed to identify factors influencing drivers’ perceived quality of VMS, which consists of 17 questions classified into four sections (S1 File). Section I is related to socio-demographic characteristics (i.e., gender, age, driving experience and driver type), in which drivers are classified by three modes: private car, office car and taxi. Particularly, the term “office car” generally refers to vehicles owned or contracted by government agencies, institutions, and state-owned enterprises for their staff to perform official duties. The variable definitions under this section are listed in Table 1.

**Table 1:** Definitions of socio-demographic characteristics.

Section	Variable	Description	Code definition
**Section I**	Gender	Male	1
		Female	2
	Age	Less than 25-year old	1
		26~35 years old	2
		36~45 years old	3
		More than 46-year old	4
	Driving experience	Less than 2 years	1
		2~5 years	2
		6~10 years	3
		More than 11 years	4
	Driver type	Private car driver	1
		Office car driver	2
		Taxi driver	3

Section II addresses questions on travel-related characteristics. For this section, respondents are asked to rate the weekly frequency of driving from one place to another into five levels, including “never”, “rarely”, “occasionally”, “very often” and “always”.

Section III is related to attitude towards the formats of VMS. The drivers are asked to express their attitude towards the format of information, including text-only, graph-only, and text-graph. In addition, attitude towards the contents of VMS, including length of congested road, travel time, reduced travel time and increased travel distance of detour routes, are considered. The attitude is ranked on a five-point scale, where 1 means strongly dissatisfied and 5 means strongly satisfied.

In Section IV, decision-making is regarded by whether VMS has ever changes drivers departure time and/or route choice, which is classified into three levels: “never”, “possible change”, and “change”. In addition, the perception of VMS information and service quality are ranked on a five point scale (strongly disagree = 1; disagree = 2; neutral = 3; agree = 4; strongly agree = 5) towards three aspects “intelligibility”, “accuracy” and “usefulness”. The variable definitions related to questions in Sections II, III and IV are listed in Table 2.

**Table 2:** Definitions of travel-related characteristics and attitude towards VMS.

Section	Dimension	Description	Code Description
**Section II**	Driving frequency	Weekly driving frequency from one place to another.	1→Never 2→Rarely 3→Occasionally 4→Very often 5→Always
**Section III**	Attitude towards formats of VMS message	Text-only	1→Strongly dissatisfied 2→Dissatisfied 3→Neutral 4→Satisfied 5→Strongly satisfied
		Graph-only	
		Text-graph	
	Attitude towards contents of VMS message	Length of congested road sections	
		Travel time	
		Saved travel time caused by detouring	
		Increased distance caused by detouring.	
**Section IV**	Driver decision-making	Change your departure time	1→Never 2→Possible change 3→Change
		Change to alternative route	
	Effectiveness of VMS message	Intelligible	1→Strongly disagree 2→Disagree 3→Neutral 4→Agree 5→Strongly agree
		Accurate	
		Useful	
	Perceived quality of service	VMS technology is up to expectation	
		VMS system is satisfactory	

### Survey implementation

The on-site survey was performed because of the high questionnaire return rate. The on-site survey is a fact-of-fact interview, so the probability of completing the survey is high. Meanwhile, surveyors randomly selected not only the local drivers but also out-of-town drivers who was traveling through the surveyed districts, which could avoid the shortage of the low representative of the sample. In order to obtain more reliable drivers perception data, an on-site survey was therefore conducted to private car drivers, office car drivers and taxi drivers in this study.

**Fig 1:**
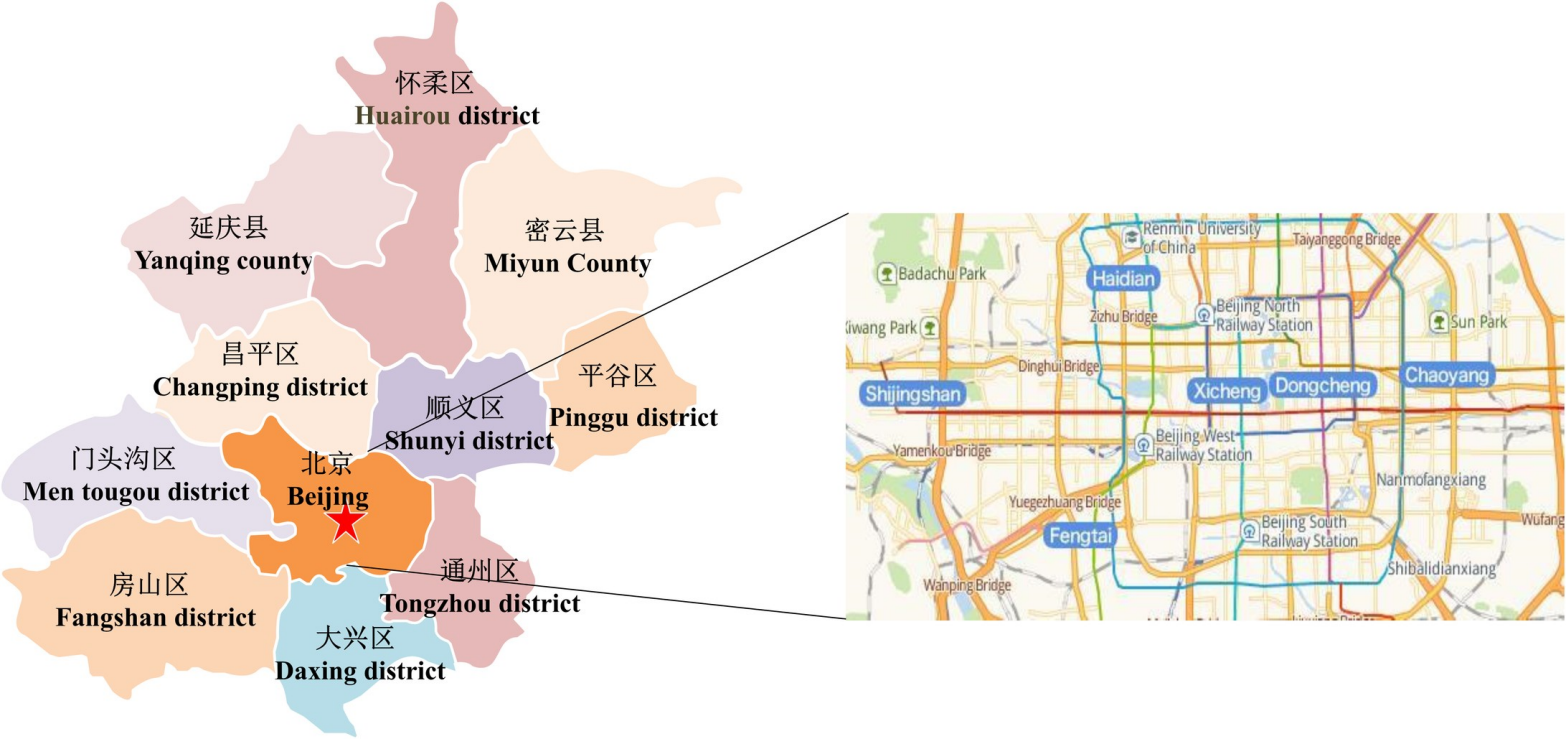
Survey area.

The questionnaires were distributed to a total of 9,600 drivers at public parking lots covering 6 districts in Beijing (e.g., Dongcheng district, Xicheng district, Haidian district, Chaoyang district, Fengtai district and Shijingshan district) (See Fig 1). There were 9,242 returned questionnaires (96.27% return rate), in which 8477 of them were valid (e.g., 5,137 from private car drivers, 1,906 from office car drivers and 1,437 from taxi drivers).

### Multiple indicators multiple causes (MIMIC) model

The MIMIC model was first proposed by Goldberger (1972) [29, 30], and then Gertler (1988) extended the model well and obtained ideal empirical results [31]. Actually, it is a measurement model with observed covariates, which can be used to study not only the relationships between latent variables and observed covariates, but also measurement invariance and population heterogeneity [32]. The MIMIC model has no strict constraints and allows measurement errors between exogenous variables and endogenous index variables, so it is more flexible than other indirect measurement methods [33]. The model generally consists of a measurement model and a structure model. The measurement model describes the relations between latent variables and indicator variables, while the structure model indicates the relations between latent variables and causal variables. The MIMIC model was formulated by Rose & Spiegel (2011) [34]:

$$y_{i,j}=\beta_{j}\xi_{i}+\upsilon_{i}$$ (1)

$$\xi_{i}=\gamma_{k}x_{i,k}+\zeta_{i}$$ (2)

where *y*_*i*,*j*_ is an observation of indicator *j* of construct *i* that represents the measurement of the construct; *β*_*j*_ is an estimated coefficient associated with indicator *j*; *ξ*_*i*_ is a latent variable of construct *i*; *υ*_*i*_ is the error term of the *i*th latent variable and casual variables; *γ*_*k*_ is an estimated coefficient of covariate *k*; *x*_*i*,*k*_ is an observation associated with covariate *k* that affecting construct *i*, representing the cause of construct *i*; *ζ*_*i*_ is the error term the *i*th latent variable and covariates.

In Eq (1), we specify *ξ*_*i*_ (*i* = 6) to include driving frequency, attitude towards formats of VMS, attitude towards contents of VMS, drivers’ decision-making, effectiveness of VMS message, and perceived quality of service. In Eq (2), we define *x*_*i*,*k*_ (*k* = 3) to include age, gender, and driving experience.

### Research hypotheses

**Fig 2:**
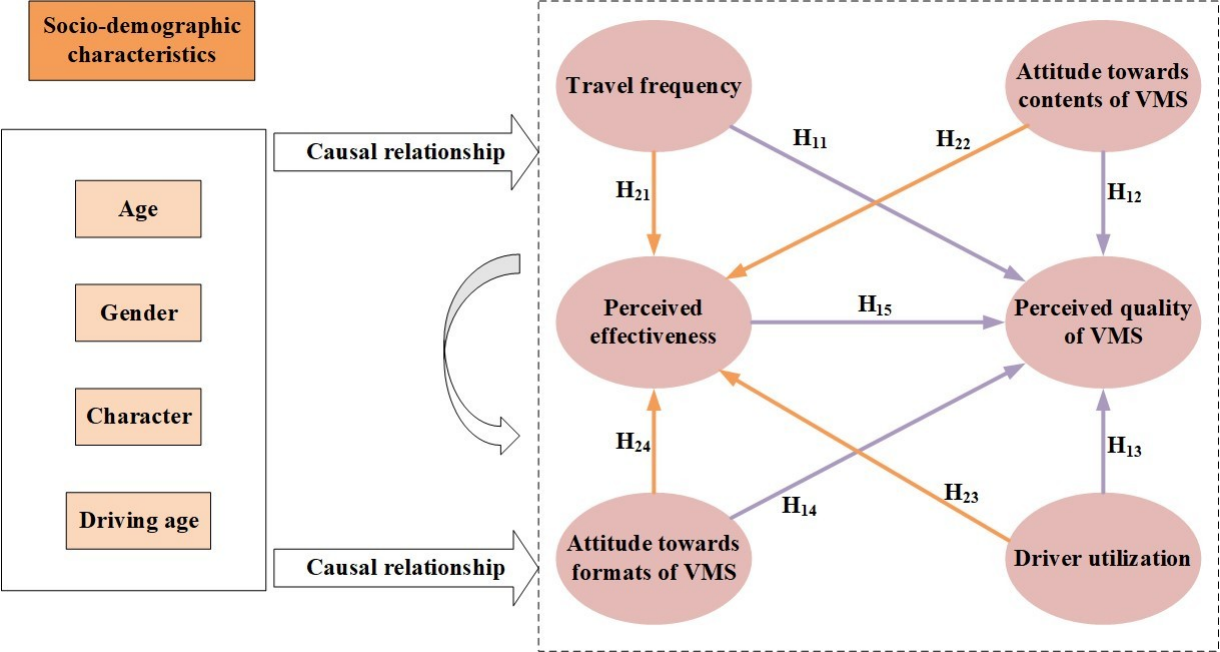
Framework and research model.

The perceived quality of service can be treated as a latent variable since it cannot be directly observed but can be inferred by a set of variables which can be either determined or measured, including driving frequency, attitude towards formats of VMS, attitude towards contents of VMS, driver decision-making, and effectiveness of VMS message. In general, drivers with high travel frequency believe that VMS is effective and then tend to have positive perception of VMS. If the drivers are satisfied with the formats and contents of VMS, they are likely to recognize the effectiveness of VMS and therefore are positive about the quality of VMS. The high utilization level (i.e. drivers are more inclined to change routes or times after receiving relevant traffic information) also means that drivers are convinced of the information provided by the VMS system and are more willing to adjust their itinerary. If drivers believe that VMS is more effective, they likely believe that the VMS service can yield their needs. Therefore, the following hypotheses are put forward (See purple lines with an arrow in Fig 2):

H_11_: Driving frequency directly affects perceived quality of service.
H_12_: Attitude towards formats of VMS directly affects perceived quality of service.
H_13_: Attitude towards contents of VMS directly affects perceived quality of service.
H_14_: Drivers’ decision-making directly affects perceived quality of service.
H_15_: Effectiveness of VMS message directly affects perceived quality of service.

Besides, these latent variables are somehow affecting each other and thus might indirectly affect the perceived quality of service. It is of importance to investigate these relations using the proposed MIMIC model. The effectiveness of VMS message is assumed to be an intermediary variable, which would directly affect perceived quality of service, but also allow other factors to indirectly affect perceived quality of service through it. Therefore, the following hypotheses are also tested (See orange lines with arrow in Fig 2):

H_21_: Driving frequency indirectly affects perceived quality of service via effectiveness of VMS message.
H_22_: Attitude towards formats of VMS indirectly affects perceived quality of service via effectiveness of VMS message.
H_23_: Attitude towards contents of VMS indirectly affects perceived quality of service via effectiveness of VMS message.
H_24_: Drivers’ decision-making indirectly affects perceived quality of service via effectiveness of VMS message.

This paper further propose that several socio-demographic characteristics such as age, gender and driving experience are regarded as control variables significant affecting latent variables (See bold arrows in Fig 2).

To verify the stability of the hypothetical model structure across driver types, the hypothetical framework structure is used for all drivers, and by analyzing each driver type separately, it is possible to compare differences across drivers specifically. Driver types are defined according to the type of vehicle used by drivers for daily travel, which can be divided into private car drivers, office car drivers and taxi drivers.

## Results

### Descriptive statistics

According to the collected data, it was found that the number of male drivers is greater than female drivers. For office cars and taxis as shown in Table 3, male drivers account for 81.7% and 84.1%, respectively, which is relatively larger than that of private cars (67.7%). Most of private car drivers are under the age of 46 (90.2%), while 71.4% of office car drivers are aged 26–45 and 70.0% of taxi drivers are over 36 year old, which indicates that middle-aged and young people prefer to drive private cars, and most of office car drivers are middle-aged people, while most of taxi drivers are middle-aged and older people. The possible reason is that some government agencies require office car drivers to be between 26 and 45 years old. Taxi driver is a hard job with low income and long working hours, so young people do not want to drive a taxi. In brief, the age distribution of drivers is different due to the types of drivers. The driving experience of majority private car drivers is less than 10 years, while office car and taxi drivers seem more experienced (e.g., almost 30% of them have greater 10 years’ experience). This may be related to the professional attribute of the driver. Due to the particularity of the profession, office car and taxi drivers are usually required to be experienced drivers, while drivers of private cars do not have such requirements.

**Table 3:** Descriptive statistics of socio-demographic characteristics.

Item	Classification	Private car drivers (%)	Office car drivers (%)	Taxi drivers (%)
Gender	Male	67.7%	81.7%	84.1%
	Female	32.3%	18.3%	15.9%
Age	≤25	23.1%	10.0%	10.8%
	26–35	41.3%	35.0%	19.2%
	36–45	25.8%	36.4%	38.2%
	≥46	9.8%	18.6%	31.8%
Drivers’ experience	<2 years	28.0%	12.5%	12.3%
	2–5 years	31.0%	20.5%	22.7%
	6–10 years	31.9%	38.0%	31.8%
	>10 years	9.1%	30.0%	33.2%

**Fig 3:**
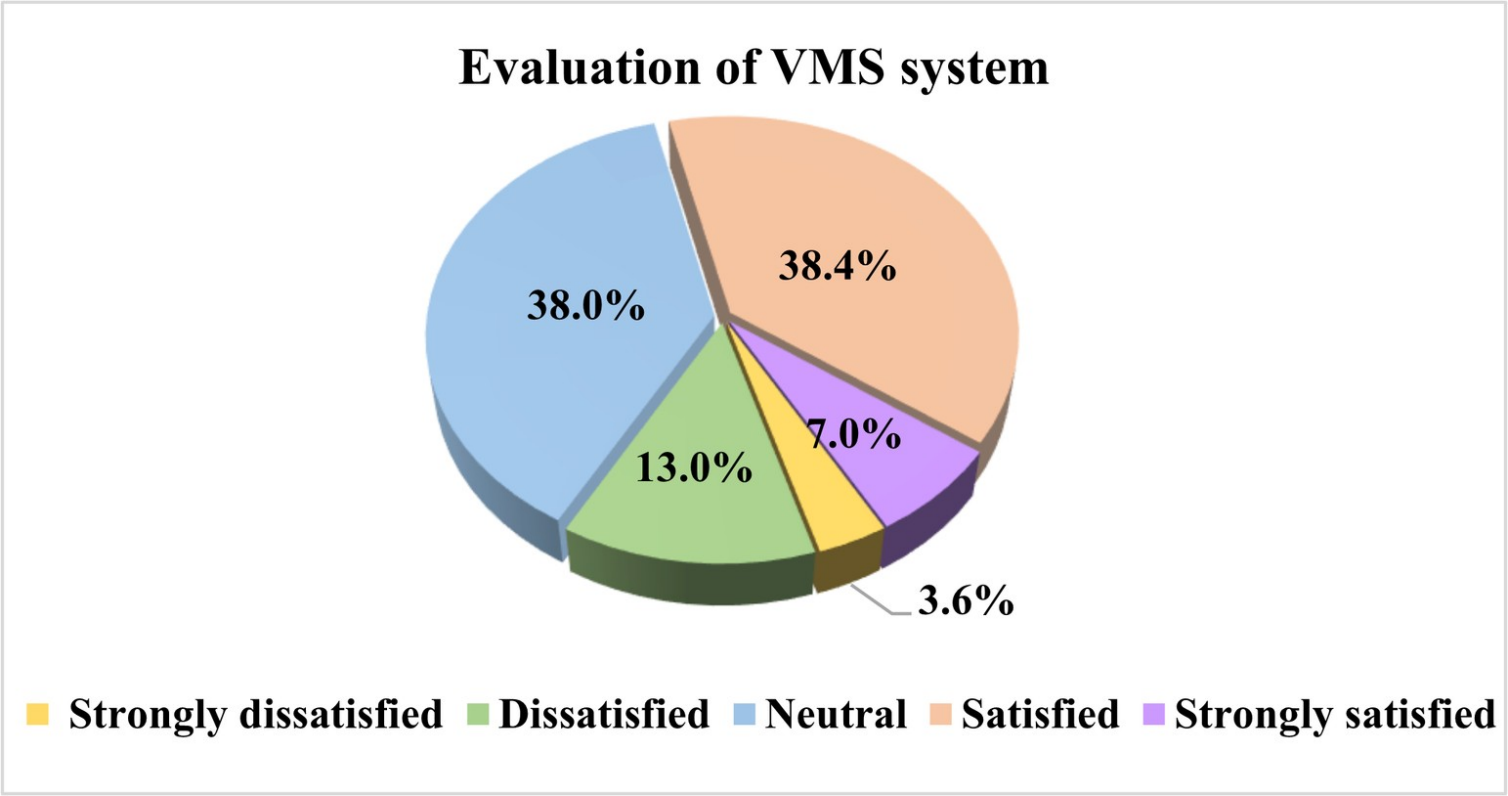
Proportional distribution of perceived quality of service.

**Fig 4:**
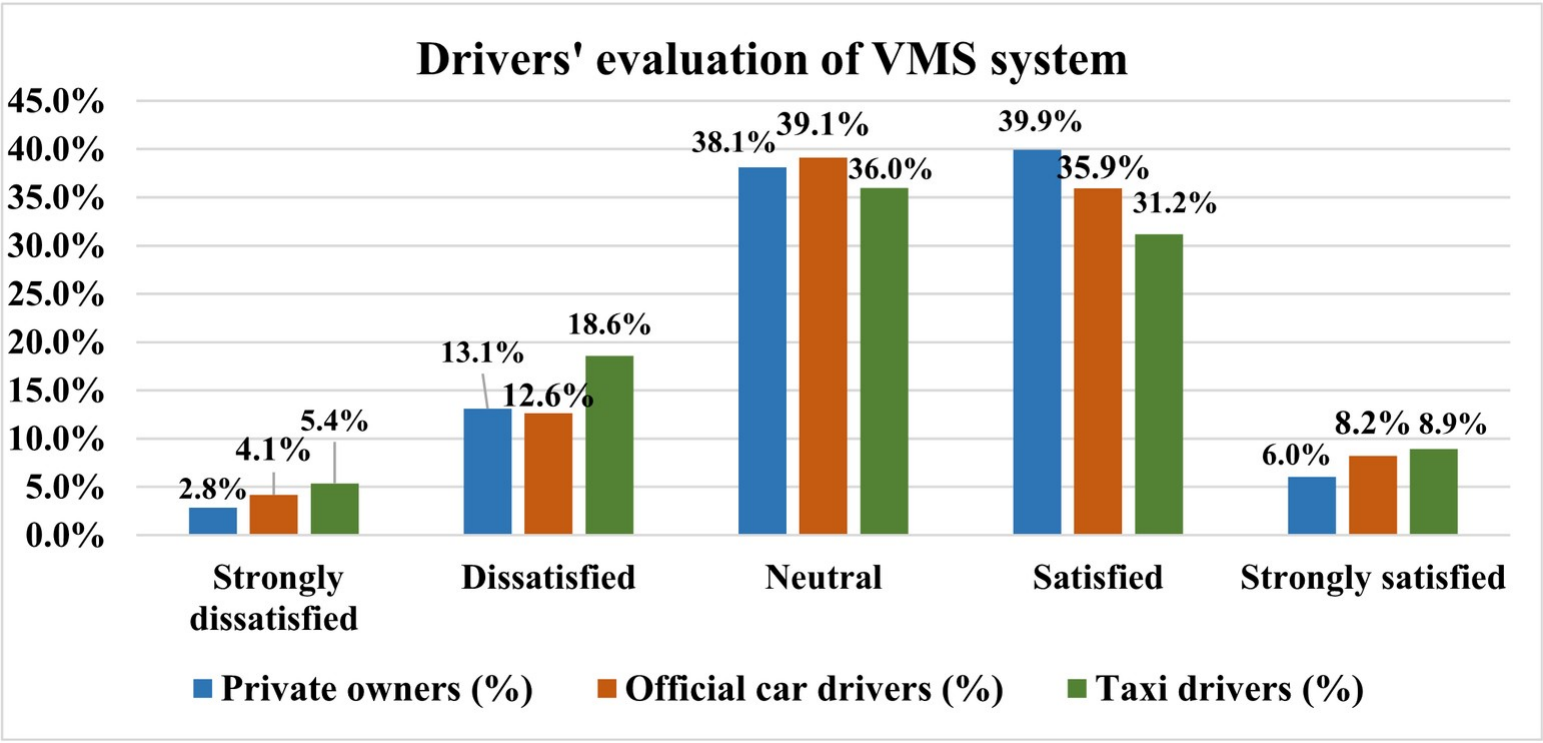
Proportional distribution of perceived quality of service by different types of drivers.

After analyzing the collected data, we found that the percentages of drivers who are satisfied, dissatisfied, and neutral with the existing VMS are 45.4%, 16.6%, and 38.0%, respectively (See Fig 3). The overall satisfaction rate is not high. Interestingly, taxi drivers responded the highest dissatisfaction rate (24.0%), which is consistent with the findings concluded by Zhou et al [45], indicating that taxi drivers seem favorable to make travel decisions based on the VMS information (See Fig 4). Private car drivers yielded the highest VMS rate (45.9%), followed by office car drivers (44.1%) and taxi drivers (40.1%). Drivers with a neutral attitude account for 38.1%, 39.1% and 36.0% of the three types of drivers respectively, indicating that a considerable number of drivers have a low awareness of VMS and have no clear judgment of the quality of VMS system.

### Reliability and validity test

In order to test the reliability and validity of the supposed latent relationship between items, the confirmatory factor analysis (CFA) is employed. The reliability measures included Canbrach’s α and construct reliability (CR), while the validity measure is average variance extracted (AVE). Canbrach’s α is a coefficient applied to evaluate the internal consistency of latent factors, which increases as the correlations between items increase. CR is used to measure of reliability and internal consistency of the measured variables representing a latent construct. Additionally, AVE is calculated to test the validity of the values, which reflects the predictive interpretation ability of the observed variable to the latent variable. The values of Canbrach’s α, CR and AVE are shown in Table 4.

**Table 4:** Results of CFA.

Driver type	Latent variable	Conbrach’s α	CR	AVE
**Private car drivers**	Driving frequency	1	1	1
	Attitude towards formats of VMS	0.721	0.739	0.743
	Attitude towards contents of VMS	0.712	0.726	0.809
	Driver decision-making	0.877	0.789	0.814
	Effectiveness of VMS message	0.731	0.765	0.803
	Perceived quality of service	0.702	0.835	0.724
**Office car drivers**	Driving frequency	1	1	1
	Attitude towards formats of VMS	0.802	0.733	0.711
	Attitude towards contents of VMS	0.724	0.748	0.824
	Driver decision-making	0.702	0.754	0.724
	Effectiveness of VMS message	0.708	0.671	0.791
	Perceived quality of service	0.801	0.865	0.792
**Taxi drivers**	Driving frequency	1	1	1
	Attitude towards formats of VMS	0.718	0.639	0.761
	Attitude towards contents of VMS	0.756	0.779	0.841
	Driver decision-making	0.834	0.765	0.811
	Effectiveness of VMS message	0.701	0.679	0.772
	Perceived quality of service	0.753	0.721	0.802

From Table 4, all Canbrach’s α are greater than 0.701, indicating that the reliability of these variables can be accepted [35]. All CR values are above 0.6 (range from 0.639 to 1.000), reflecting a good internal consistency [36]. The minimum AVE is larger than 0.5 (e.g. 0.711), indicating that the constructs have high validity [37]. Since all parameters exceed their respective critical values, the developed MIMIC model is reliable, and the explored relations among latent variables are statistically significant.

### Parameter estimation and hypothesis testing

**Fig 5:**
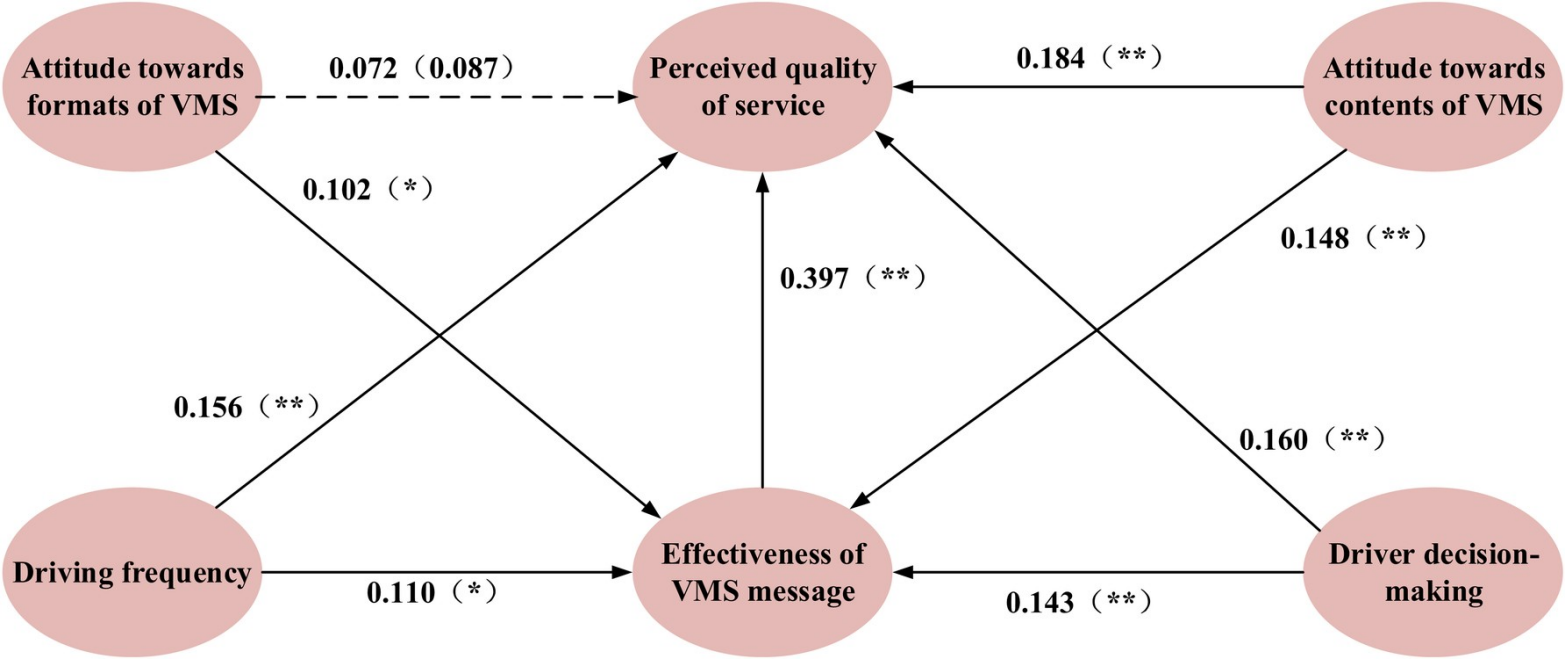
Estimated results of MIMIC for private car drivers (Model I). Notes: Coefficients are statistically significant at the 5% level (⁎ p < 0.05, ⁎⁎ p < 0.01).

**Fig 6:**
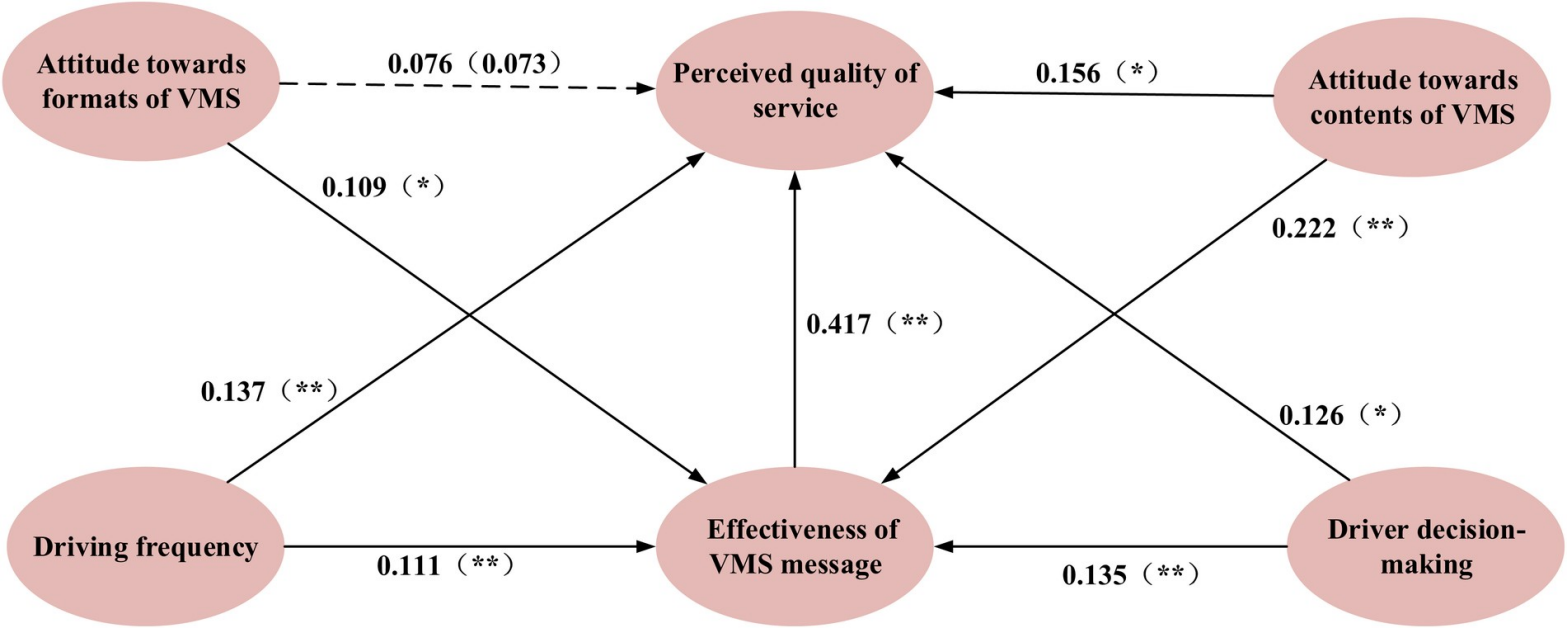
Estimated results of MIMIC model for office car owners (Model II). Notes: ** Coefficients are statistically significant at the 5% level (⁎ p < 0.05. ⁎⁎ p < 0.01).

**Fig 7:**
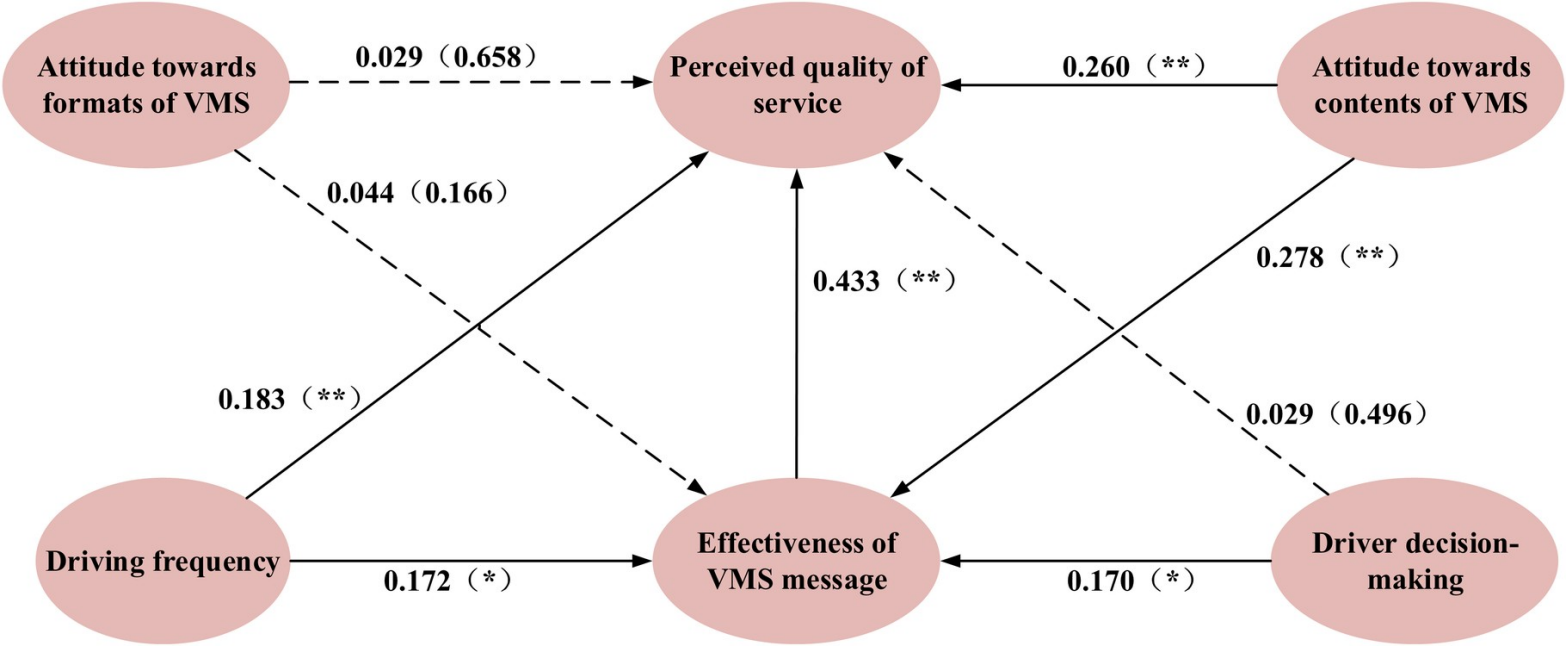
Estimated results of MIMIC model for taxi drivers (Model III). Notes: ** Coefficients are statistically significant at the 5% level (⁎ p < 0.05, ⁎⁎ p < 0.01).

To explore the factors affecting driver’s perception of VMS quality, MIMIC is employed to examine the causal relationships between potential variables and socio-demographic characteristic, and the path relations among latent variables. Considering the diversity of driver groups, it is necessary to establish three models for private car drivers, office car drivers and taxi drivers respectively. The estimated results of causal analysis summarized according to different driver categories are shown in Table 5 and the estimated path relations are shown in Figs 5–7, respectively.

**Table 5:** The estimation results between socio-demographic characteristics and latent variables.

Driver type	Latent variables	Gender	Age	Driving experience
**Private car driver**	Driving frequency	-0.171(*)	0.025(0.433)	0.387(**)
	Attitude towards formats of VMS	0.143(**)	0.032(0.152)	-0.194(*)
	Attitude towards contents of VMS	0.554(**)	0.069(0.132)	-0.409(**)
	Driver decision-making	-0.385(**)	-0.014(0.434)	0.420(**)
	Effectiveness of VMS message	0.105(*)	0.071(0.143)	-0.149(*)
	Perceived quality of service	0.165(**)	0.007(0.210)	-0.264(**)
**Office car driver**	Driving frequency	-0.119(0.541)	0.329(**)	0.294(*)
	Attitude towards formats of VMS	-0.093(0.323)	-0.017(0.542)	-0.506(**)
	Attitude towards contents of VMS	-0.029(0.288)	-0.076(0.145)	-0.427(**)
	Driver decision-making	0.605(**)	-0.015(0.231)	0.314(**)
	Effectiveness of VMS message	0.113(0.145)	-0.119(0.221)	-0.171(*)
	Perceived quality of service	0.062(0.246)	-0.065(0.435)	-0.355(**)
**Taxi driver**	Driving frequency	-0.087(0.225)	-0.031(0.281)	0.497(0.122)
	Attitude towards formats of VMS	-0.011(0.681)	-0.027(0.461)	-0.034(0.314)
	Attitude towards contents of VMS	0.082(0.155)	-0.097(0.214)	-0.008(0.782)
	Driver decision-making	-0.411(**)	0.033(0.322)	0.301(**)
	Effectiveness of VMS message	-0.029(0.554)	-0.232(**)	-0.261(*)
	Perceived quality of service	0.017(0.525)	-0.319(**)	-0.442(**)

Note: The values in brackets are p-values. The significant variables require the corresponding p-value to be less than 0.05 at the 95% confidence interval (⁎ p < 0.05, ⁎⁎ p < 0.01).

As illustrated Fig 5, four variables, including travel frequency (coefficient = 0.156, p < 0.01), attitude towards contents of VMS (coefficient = 0.184, p < 0.01), decision-making (coefficient = 0.16 0, p < 0.01) and perceived effectiveness (coefficient = 0.397, p < 0.01), directly affect perceived quality of service, while attitude towards formats of VMS (coefficient = 0.072, p = 0.087) does no. Therefore, the hypotheses H_11_, H_13_, H_14_ and H_15_ are accepted, but H_12_ is rejected. Besides, driving frequency (coefficient = 0.110, p < 0.05), attitude towards formats of VMS (coefficient = 0.102, p < 0.05), attitude towards contents of VMS (coefficient = 0.148, p < 0.01) and drivers’ decision-making (coefficient = 0.143, p < 0.01) indirectly affect drivers’ perceived quality of service via effectiveness of VMS message. Hence, H_21_, H_22_, H_23_ and H_24_ are accepted. In conclusion, there are three of the five variables, namely driving frequency, attitude towards contents of VMS and drivers’ decision-making, having both direct and indirect effects on perceived quality of service, while attitude towards formats of VMS only indirectly influence perceived quality of service. Effectiveness of VMS message not only directly influence perceived quality of service, but also allow other four variables to indirectly affect perceived quality of service via it.

From Fig 6, there are four variables, namely driving frequency (coefficient = 0.137, p < 0.01), attitude towards contents of VMS (coefficient = 0.156, p < 0.05), drivers’ decision-making (coefficient = 0.126, p < 0.05) and effectiveness of VMS message (coefficient = 0.417, p < 0.01), directly affecting perceived quality of service, while attitude towards formats of VMS (coefficient = 0.076, p = 0.073) does no. Therefore, the hypotheses H_11_ H_13_, H_14_, and H_15_ are accepted, but H_12_ is rejected. In addition, driving frequency (coefficient = 0.111, p < 0.01), attitude towards formats of VMS (coefficient = 0.109, p < 0.05), attitude towards contents of VMS (coefficient = 0.222, p < 0.01) and driver decision-making (coefficient = 0.135, p < 0.01) indirectly influence perceived quality of service via effectiveness of VMS message, hence H_21_, H_22_, H_23_ and H_24_ are accepted. To sum up, there are three of the five variables, namely driving frequency, attitude towards contents of VMS and drivers’ decision-making, having both direct and indirect effects on perceived quality of service, while attitude towards formats of VMS only indirectly influence perceived quality of service. Effectiveness of VMS message not only directly influence perceived quality of service, but also allow other four variables to indirectly affect perceived quality of service via it.

From Fig 7, driving frequency (coefficient = 0.183, p < 0.01), attitude towards contents of VMS (coefficient = 0.260, p < 0.01) and effectiveness of VMS message (coefficient = 0.433, p < 0.01) can influence perceived quality of service, while attitude towards formats of VMS (coefficient = 0.029, p = 0.658) and drivers’ decision-making (coefficient = 0.029, p = 0.496) do not. Therefore, the hypotheses H_11_, H_13_, H_15_ are accepted, but H_12_ and H_14_ are rejected. In addition, driving frequency (coefficient = 0.172, p < 0.05), attitude towards contents of VMS (coefficient = 0.278, p < 0.01) and drivers’ decision-making (coefficient = 0.170, p < 0.05) indirectly affected driver’s perceived quality of service via effectiveness of VMS message, thus H_21_ H_23_ and H_24_ are accepted. However, H_22_ is rejected because attitude towards formats of VMS (coefficient = 0.044, p = 0.166) has no indirect effect on driver’s perceived quality of service via effectiveness of VMS message. To sum up, there are two of the five variables, namely driving frequency and attitude towards contents of VMS, having both direct and indirect effects on perceived quality of service, while attitude towards formats of VMS has no effect on perceived quality of service, drivers’ decision-making only indirectly influence perceived quality of service. Effectiveness of VMS message not only directly influence perceived quality of service, but also allow other four variables to indirectly affect perceived quality of service via it.

The hypothesis test results of the three models are shown in Table 6.

**Table 6:** Results of hypothesis testing.

Hypotheses	Model I	Model II	Model III
H_11_: Driving frequency → Perceived quality of service	Accept	Accept	Accept
H_12_: Attitude towards formats of VMS →Perceived quality of VMS	Reject	Reject	Reject
H_13_: Attitude towards contents of VMS → Perceived quality of service	Accept	Accept	Accept
H_14_: Drivers’ decision-making → Perceived quality of service.	Accept	Accept	Reject
H_15_: Effectiveness of VMS message→ Perceived quality of service	Accept	Accept	Accept
H_21_: Driving frequency →Effectiveness of VMS message.	Accept	Accept	Accept
H_22_: Attitude towards formats of VMS → Effectiveness of VMS message	Accept	Accept	Reject
H_23_: Attitude towards contents of VMS → Effectiveness of VMS message	Accept	Accept	Accept
H_24_: Drivers’ decision-making →Effectiveness of VMS message	Accept	Accept	Accept

In summary, there are some differences and commonalities in estimated results of model among three type of drivers. What they have in common is that driving frequency, attitude towards contents of VMS and effectiveness of VMS message can directly affect driver’s perceived quality of service and attitude towards formats of VMS has no direct effect on perceived quality of service for the three type of drivers. In addition, driving frequency, attitude towards contents of VMS and drivers’ decision-making can also indirectly influence perceived quality of service through effectiveness of VMS message. The difference is that the drivers’ decision-making can directly affect the perceived quality of service for private car drivers and office car drivers, but there is no impact for taxi drivers. Also, attitude towards formats of VMS can indirectly impact on perceived quality of service through effectiveness of VMS message for private car drivers and office car drivers, while there is no indirect impact for taxi drivers.

Five goodness-of-fit indicators, including the ratio of chi-square to the degree of freedom (*χ*^2^/*d*_*f*_), comparative fit index (CFI), tucker-lewis index (TLI), root mean square error of approximation (RMSEA) and root mean residual (RMR), are selected to test the three developed models. The goodness-of-fit test results of all models are presented in Table 7.

**Table 7:** Goodness-of-fit test results.

Indicator	Criteria	Model I	Model II	Model III
*χ*^2^/*d*_*f*_	<3	2.506	2.287	2.811
CFI	>0.9	0.913	0.929	0.911
TLI	>0.9	0.903	0.951	0.925
RMSEA	<0.08	0.069	0.063	0.079
RMR	<0.08	0.078	0.077	0.078

From Table 7, *χ*^2^/*df* of three models is 2.506, 2.287 and 2.811, respectively, which are less than the standard threshold of 3, indicating that indicator is acceptable [38–41]. CFI and TLI all exceed the acceptable threshold of 0.9 [38]. Other fitting indicators, such as RMR and RMSEA, are also found to be acceptable by model fit criteria [42]. On the whole, all goodness-of-fit indices of the three models meet the suggested criteria, indicating that all models obtained are statistically appropriate, and the hypothetical framework model is therefore suitable for analyzing driver’s perceived quality of service.

## Discussion

### Analysis of the relationship between socio-demographic characteristics and potential variables

As seen from Table 5, gender and driving experience have a significant effect on driving frequency of private car drivers (coefficient = -0.171, p < 0.05; coefficient = 0.387, p < 0.01), suggesting well experienced male private owner travel more frequently. the gender of private car drivers positively affects their attitude towards formats and contents of VMS (coefficient = 0.143, p < 0.01; coefficient = 0.554, p < 0.01), while driving experience has a negative effect on their attitude towards formats and contents of VMS (coefficient = -0.194, p < 0.05; coefficient = -0.409, p < 0.01), which indicated that female drivers with little driving experience tend to be satisfied with the formats and contents of VMS. This may be related to the fact that inexperienced female drivers have higher demand for traffic information than men [43]. Gender negatively influence driver decision-making (coefficient = -0.385, p < 0.01), while driving experience positively affects driver decision-making (coefficient = 0.420, p < 0.01), indicating that well experienced and female private car owner is inclined to change their travel decisions. This result is supported by previous studies [3, 44]. In addition, gender positively affect private car drivers’ perceived effectiveness and quality of VMS (coefficient = 0.105, p < 0.05; coefficient = 0.165, p < 0.01), while driving experience has a negative impact on the perceived effectiveness and quality and of VMS (coefficient = -0.149, p < 0.05; coefficient = -0.264, p < 0.01). This result suggests that female and less experienced drivers can perceive the effectiveness of VMS and believe that the traffic information provided by VMS is necessary and accurate, which may because that female drivers are more conservative and risk-conscious than male drivers, so they tend to rely on and trust traffic information provided by VMS to ensure traffic safety [45].

For office car drivers, age and driving experience positively influence driving frequency (coefficient = 0.329, p < 0.01; coefficient = 0.294, p < 0.05), suggesting male office car drivers with well driving experience tend to have a high driving frequency. One possible explanation is that skilled and experienced drivers are safer drivers, worthy of the trust of the company’s bosses, and thus have more opportunities to travel. Similar to private car drivers, driving experience of office car driver negatively affect their attitude towards formats and contents of VMS (coefficient = -0.506, p < 0.01; coefficient = -0.427, p < 0.01). This may be related to the fact that inexperienced office car drivers have higher demand for traffic information than well experienced ones [45], hence they are satisfied with the formats and contents of VMS. In addition, the drivers’ decision-making of office car drivers is affected by gender and driving experience (coefficient = 0.605, p < 0.01; coefficient = 0.314, p < 0.01), a finding which confirms the conclusion reached by Zhao et al [23], who concluded that less experienced and young drivers are more likely to divert to an alternative route. Driving experience has a negative impact on the perceived effectiveness and quality of VMS (coefficient = -0.171, p < 0.05; coefficient = -0.355, p < 0.01), implying that office car drivers with less driving experience often think that the information provided by VMS is accurate, reliable and necessary for travelers.

As for taxi drivers, there is no factor affecting driving frequency and drivers’ attitude towards formats and contents of VMS, which may be related to the nature of taxi drivers’ work. According to Zhou et al (2005) conclusion, taxi drivers are generally male, with driving experience for about 10 years a higher familiarity with the road network [45]. Therefore, the statistical correlation between the factors in this paper is not significant. Gender and driver experience have an effect on drivers’ decision-making (coefficient = -0.411, p < 0.01; coefficient = 0.301, p < 0.01), which is consistent with Zhou et al (2005), who concluded that well experienced male taxi drivers are more familiar with the road network, hence they are more inclined to rely on their own experience to make travel decisions [44]. In addition, age negatively affect taxi drivers’ perceived effectiveness and quality of VMS (coefficient = -0.232, p < 0.01; coefficient = -0.319, p < 0.01), and driving experience also has a negative impact on the perceived effectiveness and quality of VMS (coefficient = -0.261, p < 0.05; coefficient = -0.442, p < 0.01).

### Discussion on influencing factors of drivers’ perceived quality of service

From the structural models of the three driver categories developed in the study, it is obvious that the perceived quality of service cannot be evaluated independently of the driver’s driving frequency, attitude towards the formats and contents of VMS, drivers’ decision-making and effectiveness of VMS message. All the factors assumed in this study contribute directly or indirectly to the assessment of perceived service of quality. The relationship between determinants and perceived quality of service is discussed as follow.

One the one hand, as for private car drivers, office car drivers and taxi drivers, driving frequency, attitude towards contents of VMS and effectiveness of VMS message can directly affect perceived quality of service, and attitude towards formats of VMS has no direct effect on perceived quality of service. First, driving frequency directly affects perceived quality of service (H_11_), a finding which confirms the conclusion reached by Khoo and Ong (2013) [6], and one possible reason is that drivers who travel frequently need to obtain more information about their trip either pre-trip or en-route in order to enjoy smoother rides. Second, attitude towards contents of VMS also directly affects perceived quality of service (H_13_). This can perhaps be explained that travelers’ satisfaction with the contents of the VMS indicates that the service level of the VMS system is acceptable, and thus higher satisfaction may lead to higher perceived quality of service. Third, effectiveness of VMS message is the most important factor that affects driver’s perceived quality of service (H_15_), a finding which confirms the conclusion reached by Khoo and Ong (2013), who concluded that a better perception of ITIS system effectiveness, accuracy and reliability can well indicate a high perceived quality of service [6]. Attitude towards formats of VMS has no direct effect on perceived quality of service (H_12_) and this may because that drivers attach importance to the contents released by the VMS system and the detail of the contents, but pay less attention to the format of contents presentation.

Besides, driving frequency, attitude towards contents of VMS and drivers’ decision-making can indirectly affect driver’s perceived quality of service for the three driver types (H_21_, H_23_, H_24_). This result suggests that drivers who have high driving frequency and are satisfied with the contents of VMS system can better perceive the intelligibility, accuracy and usefulness of VMS system, thus resulting in higher perceived quality of service. Besides, driver who make route changes under the real-time traffic information provided by VMS generally think that the information is reliable and accurate, and then VMS system is considered to have higher service quality.

On the other hand, there are some differences between the three types of drivers. The drivers’ decision-making directly affect the perceived quality of service for private car drivers and office car drivers(H_14_), which is supported by Khoo and Ong (2013), who concluded that if drivers are more willing to accept and utilize the information obtained, this may indicate that the VMS system is operating at a specific service level acceptable to the user [6]. But there is no impact for taxi drivers and this may be because taxi drivers are usually familiar with the road traffic due to the particularity of their profession, and they do not trust the information provided by VMS and change their travel routes [45], thus making the survey data not statistically significant. As for private car drivers and office car drivers, attitude towards formats of VMS has an indirect impact on perceived quality of service through effectiveness of VMS message (H_22_), while there is no indirect impact in the model of taxi drivers. This may be because taxi drivers usually have low dependence and cognition on VMS system, which confuses the relationship between formats of VMS and the effectiveness of VMS.

## Conclusions

There is a dearth of research on evaluating the service quality of VMS system from the perspective of driver perception, therefore, this paper presents the modeling of perceived quality of service using MIMIC, which is used to explore drivers’ attitude to service quality of VMS system and identify the predominant influencing factors. To compare differences across drivers specifically and verify the stability of the hypothetical model across driver categories, the perceived quality of service is analyzed for private car drivers, office car drivers and taxi drivers, respectively.

The research results show that the proposed hypothetical model allows a holistic interpretation on perceived quality of service. Through the analysis of the relationship between socio-economic characteristics and latent variables, it is found that gender, age and driving experience have significant effects on latent variables. Driving experience is the most important factor, followed by gender. Driving frequency, attitude towards contents of VMS, and effectiveness of VMS message can direct and indirectly affect driver’s perceived quality of service and attitude towards formats of VMS has no direct effect on perceived quality of service for the three driver types. The drivers’ decision-making directly affects the perceived quality of service for private car drivers and office car drivers, but there is no impact for taxi drivers. Besides, attitude towards formats of VMS indirectly impact on perceived quality of service through effectiveness of VMS message, while there is no indirect impact for taxi drivers.

Based on the results and contributions, some recommendations to improve the VMS system are put forward. First of all, it is necessary to develop smart phone applications considering personal demands that would allow users to obtain real time traffic information. Secondly, it is important to optimize the content provided by VMS, such as providing advice on the best departure time and routes to choose, providing predicted travel times and delays on VMS messages. Last but not the least, it is necessary to improve the quality of service, such as providing more reliable and accurate travel time by VMS, and improving VMS coverage to include traffic conditions on alternate routes. In summary, the research can provide some insights to future VMS planning, design and operation in Beijing and thus has potential to improve the usage efficiency and service quality of VMS based on the available resources and design effective in-vehicle advanced driver assistance systems.

The following limitations need to be noted and addressed in future research.

First, there are some areas of development to be considered in designing and implementing the questionnaire. The questionnaire included no open-ended questions, leading to the phenomenon that participants may be forced to choose an option that does not fully represent their real opinion, and little feedback as to how the VMS system might be improved in the future. Besides, there is only one driving frequency for the driver’s travel characteristic variables. Other variables, such as travel distance and travel purpose, can be considered in the future study.

Then, the ordinal data we collected using Likert-scale items cannot be subject to addition, subtraction, means, or standard deviation calculation. This further restricts comparison or other quantitative analysis which could be done with interval or ratio data.

Finally, the purpose of this study is to develop a holistic model which can be applicable to three types of drivers, and use a MIMIC model to qualitative analyze impact on driver’s perceived service quality and interaction between variables according to different driver categories in order to explore the differences between different drivers. Therefore, one of the defects of the research is that there is no specific and quantitative analysis of the changes of independent variables on driver’s perceived service quality. In the future, the hybrid method integrating the MIMIC model and a generalized ordered response model [46, 47] will be used to explore the relations between independent variables and driver’s perceived service quality, and then elasticity analysis will also be adopted to study the changes of independent variables on driver’s perceived service quality.”

## Supporting information

S1 File Questionnaire on perceived service quality of VMS. (DOCX)
