# Supplementary material for: Analyzing drivers’ perceived service quality of variable message signs (VMS)
Source: PLoS One. 2020 Oct 21;15(10):e0239394. doi: 10.1371/journal.pone.0239394 (PMC7577472; doi:10.1371/journal.pone.0239394)
Supplement: S1 File — (DOCX) [file pone.0239394.s001.docx]

**Questionnaire on perceived service quality of variable information signs（VMS）**

Thank you for participating in this questionnaire survey! The information you fill in will be used to optimize the traffic information service and will be kept strictly confidential. Please fill in the corresponding options in brackets. Your participation will make Beijing's road traffic smoother!

Socio-demographic characteristics.

| 1. Gender | （1） Male （2） Female |
| --- | --- |
| 2. Age | （1） Less than 25-year old （2）26~35 years old （3）36~45 years old （4） More than 46-year old |
| 3. Driving experience | （1） Less than 2 years （2）2~5 years （3）6~10 years （4）More than 11 years |
| 4. Driver type | （1） Private car driver （2） Office car driver （3） Taxi driver |
| 5. Weekly driving frequency from one place to another | （1） Never （2） Rarely （3） Occasionally （4） Very often  （5） Always |

Your attitude towards formats and contents of VMS message. **Tick the appropriate option label (select only one option)**

|  | Strongly dissatisfied | Dissatisfied | Neutral | Satisfied | Strongly satisfied |
| --- | --- | --- | --- | --- | --- |
| 1. text-only format |  |  |  |  |  |
| 2. graph-only format |  |  |  |  |  |
| 3. Text-graph |  |  |  |  |  |
| 4. Length of congested road sections |  |  |  |  |  |
| 5. Travel time |  |  |  |  |  |
| 6. Saved travel time caused by detouring |  |  |  |  |  |
| 7. Increased distance caused by detouring. |  |  |  |  |  |

According to your actual situation and feelings， **please tick the appropriate option label (select only one option).**

| 1. Do you change your departure time upon receiving of traffic information?  （1）Never （2）Possible change（3）Change |
| --- |
| 2. Do you change to alternative route upon receiving congestion information?  （1）Never （2）Possible change（3）Change |
| 3. The information released by VMS is intelligible  （1）Strongly disagree （2）disagree（3）Neutral （4）Agree （5）Strongly agree |
| 4. The information released by VMS is accurate  （1）Strongly disagree （2）disagree（3）Neutral （4）Agree （5）Strongly agree |
| 5. The information released by VMS is useful  （1）Strongly disagree （2）disagree（3）Neutral （4）Agree （5）Strongly agree |
| 6. The current VMS system is good and up to your expectation  （1）Strongly disagree （2）disagree（3）Neutral （4）Agree （5）Strongly agree |
| 7. You are satisfied with the service of the current VMS system  （1）Strongly disagree （2）disagree（3）Neutral （4）Agree （5）Strongly agree |

**可变信息标志的感知服务质量调查问卷**

感谢您参与本次问卷调查！您填写的信息将用于优化交通信息服务，并会被严格保密。请您将对应的选项填入括号中。您的参与会使北京的道路交通更通畅！

一、您的个人情况。

| 1.您的性别 | （1）男 （2）女 |
| --- | --- |
| 2.您的年龄 | （1）25岁以下 （2）26-35岁 （3）36-45岁 （4）46岁以上 |
| 3.您的驾驶经验 | （1）2年以下 （2）2-5年 （3）6-10年 （4）11年以上 |
| 4.您驾驶的车辆 | （1）私家车 （2）办公用车 （3）出租车 |
| 5.您每周驾车出行的频率 | （1）从不 （2）很少 （3）偶尔 （4）经常 （5）总是 |

二、您对目前VMS发布形式和发布内容的态度。**在相应的选项标号上打“√”（只能选择一个选项）。**

|  | 非常不满意 | 不满意 | 中立 | 满意 | 非常满意 |
| --- | --- | --- | --- | --- | --- |
| 1.文字式信息发布形式 |  |  |  |  |  |
| 2.图形式信息发布形式 |  |  |  |  |  |
| 3.图文混合式信息发布形式 |  |  |  |  |  |
| 4.拥堵路段长度 |  |  |  |  |  |
| 5.到某一地点的行程时间 |  |  |  |  |  |
| 6.建议绕行线路的路况信息 |  |  |  |  |  |
| 7.建议绕行线路的行驶时间 |  |  |  |  |  |

三、请根据您的实际情况和感受，**在相应的选项标号上****打“√”（只能选择一个选项）。**

| 1.当收到拥堵信息时，您会改变出行时间吗  （1）不改变 （2）可能改变（3）改变 |
| --- |
| 2.当收到拥堵信息时，您会改变出行路线吗  （1）不改变 （2）可能改变（3）改变 |
| 3.可变信息标志发布的信息是易理解的  （1）非常不同意 （2）不同意（3）中立 （4）同意 （5）非常同意 |
| 4.可变信息标志发布的信息是准确的  （1）非常不同意 （2）不同意（3）中立 （4）同意 （5）非常同意 |
| 5.可变信息标志发布的信息是有用的  （1）非常不同意 （2）不同意（3）中立 （4）同意 （5）非常同意 |
| 6.您认为目前的VMS系统很好，达到了您的期望  （1）非常不同意 （2）不同意（3）中立 （4）同意 （5）非常同意 |
| 7.您很满意目前VMS系统的服务水平  （1）非常不同意 （2）不同意（3）中立 （4）同意 （5）非常同意 |
